# Entertainment apps, limited attention and investment performance

**DOI:** 10.3389/fpsyg.2023.1118797

**Published:** 2023-04-17

**Authors:** Yingxin Zhang, Yijing Du, Yan Li

**Affiliations:** ^1^School of Economics, Department of Finance, Beijing Technology and Business University, Beijing, China; ^2^School of Business, Department of Finance, Renmin University of China, Beijing, China

**Keywords:** limited attention, entertainment-type app, mobile age, investment, P2P

## Abstract

With the advent of the “information age,” investors are now faced with the challenges of the “mobile age,” which has had a profound impact on the daily lives of people worldwide. Investors must process more information while experiencing increasing mobile phone-related distractions, particularly those generated by the fast-growing entertainment-type app industry. Attention is a limited cognitive resource that is vital for deliberate and thoughtful analysis. We analyzed data from an online peer-to-peer lending market to evaluate the impact of mobile distractions on investment performance. Our findings revealed that investors with a large number of mobile phone entertainment apps were more likely to exhibit higher default rates and lower investment returns. The results are robust, even when using exogenous internet service outage of the entertainment server and instrumental variables. We observed that the negative impact of distraction was more pronounced on Fridays and in regions with high-speed Internet access. A further examination of the mechanisms underlying this phenomenon revealed that investment decisions made while being distracted by mobile apps were influenced by information neglect and familiarity biases.

## 1. Introduction

In the information age, people are facing an increasing demand to concentrate and perform a variety of daily tasks and challenges that require attention (Buschman and Miller, 2007). However, research shows that attentional resources are limited (Kahneman and Tversky, 1973; Falkinger, 2008). Economics researchers have examined the concept of limited attention in several contexts, such as Friday earnings announcements (DellaVigna and Pollet, 2009), marital events (Lu et al., 2016), and large jackpot lotteries (Huang et al., 2019). In present times, the excessive and problematic use of mobile phones has been widely documented.^1^ It is worth noting that mobile phones compete for limited attention through the extensive mobile applications (apps) of social networks, videos, games, and other entertainment types. Beneito and Vicente-Chirivella (2022) discovered that banning mobile phones in schools had a significant positive impact on student performance in Spain. The use of mobile phones in the classroom, despite its potential to facilitate structured learning, can also lead to distractions that negatively impact academic performance. Brown et al. (2020) used the Blackberry Internet Service outage and discovered that a higher level of information gathering and increased trading activity were associated with greater views or downloads of disclosure filings; these findings suggest that the mobile Internet has the potential to divert the attention of stock market activities. However, the extent to which entertainment apps distract individuals and hinder their decision-making abilities remains unclear. In this study, we addressed these questions by examining the number of entertainment apps installed by investors and their online peer-to-peer (P2P) investment performance.

A growing body of evidence from psychology and health research shows that the use of mobile phones has a detrimental impact on the cognitive attention required to focus on daily tasks effectively (Anderson et al., 2017; Grewal et al., 2018). Moreover, entertainment apps are among the most concerning sources of distraction (Brooks, 2015; Swar and Hameed, 2017; Chu et al., 2021). Wu et al. (2018) found that the fear of missing out drives people to constantly speculate about what their families and friends are doing on social networks and the latest popular short videos; additionally, a sense of excitement and achievement linked to passing higher levels encourages people to play mobile games. Thornton et al. (2014) postulated that simply having a visually noticeable mobile phone can evoke a sense of being excluded from a “broad social and informational network... that one is not part of at the moment,” even when people are not using their phones. Wilmer et al. (2017) claimed that smartphones can negatively impact focused and sustained attention and diminish the capacity for achieving a state of flow (Csikszentmihalyi and Larson, 2014). Extending these findings to the investment context, we suggest that the attention-diverting effects of entertainment apps may result in investors making suboptimal investment decisions.

By using the number of installed mobile entertainment apps of P2P investors to measure attention distraction, our study found that investors with a greater number of such apps have a significantly higher likelihood of default and a lower internal rate of return, even after controlling for loans, borrowers, and investor-level characteristics. We were fully aware that there could be potential omitted control variables and that a reverse causality relationship may exist when downloading more entertainment apps for leisure or in response to poor investment outcomes. To address these concerns, we used a time-varying difference-in-difference analysis based on the entertainment company's seven large-scale national Internet service outages. We found that diminishing attentional distraction shock resulted in improved performance. We also used the Baidu search index of entertainment-related app keywords in the investor's city and the average number of entertainment apps downloaded among people of the same age and gender as the investor as instrumental variables (IV), and we found that the results remained robust.

To further assess the attention-distraction effect of entertainment apps, we differentiated between Friday and other working days and high- and low-speed Internet regions. DellaVigna and Pollet (2009) discovered that Friday distracted investors from job-related tasks. Since entertainment-related apps facilitate chatting with families and friends to schedule upcoming weekends and searching for new movies and videos to enjoy at weekends, they could cause a more pronounced attention-limitation effect on Friday compared with Monday to Thursday. In addition, a smooth mobile network is essential for a better user experience, especially for entertainment-related apps that comprise a large number of videos, pictures, and real-time communication. We discovered that the impact of entertainment-related apps on limited attention is more pronounced in high-speed Internet regions because of the improved user experience. We also found consistent evidence to support the identification validity of using the number of entertainment apps as an indicator of different levels of attention distraction among P2P investors.

Regarding the underlying mechanism of poor performance as a result of entertainment apps, research on economics and finance has shown that, if attention is limited, some information has to be ignored (Peng and Xiong, 2006; Choi and Choi, 2019; Wang and Song, 2022; Zhong, 2022). Moreover, people may rely on simple mental shortcuts to process information when their attention is limited. Barber and Odean (2008) discovered that attention-grabbing stocks tend to be bought in, and Lacetera et al. (2012) showed the existence of a “left-digit” bias. Iscenko (2020) found that borrowers exposed to higher levels of distraction tend to direct their attention toward lenders with whom they have an existing personal relationship. Using data from a P2P lending market, we explored the potential mechanisms underlying investment behavior from the perspectives of information-processing ability and familiarity heuristics. The results showed that investors with higher levels of distraction, indicated by more entertainment apps, exhibit worse investment performance when faced with more readily available information and are familiar with the borrowers.

Our study adds to the literature in many ways. First, it extends the examination of the attention-distraction effect at the individual investor level. Economics researchers have used various methods to identify limited attention and have incorporated potential distractions to understand how attention is allocated to a particular event (Lu et al., 2016; Campbell et al., 2019). However, existing research mainly focuses on market-wide attention distraction and professional institutional investors. Ben-Rephael et al. (2017) pointed out that the decision to focus on retail investors, institutional investors, or both should be empirically determined because of their different roles in the finance market. Bajo et al. (2021) studied the distraction effect of birthdays and found a decrease in trading activity in a three-day window. In this study, we showed that mobile entertainment apps can cause investor attention to shift away from investment, leading to a decrease in investment performance.

Second, our study also contributes to the understanding of the underlying mechanism of inferior decision-making caused by limited attention. Previous theoretical models suggested that limited attention can explain category learning (Peng and Xiong, 2006) and style investing (Barberis and Shleifer, 2003; Wahal and Yavuz, 2013) and have important implications for other asset pricing. In this study, we attempted to empirically test whether investors neglect partial information and use heuristics when their attention is limited. Our study echoes previous studies on how limited attention affects the incorporation of information into the decision-making process. There is substantial evidence that people tend to use simple heuristics when processing information in tasks such as categorization (Mullainathan, 2002; Barberis and Shleifer, 2003), simple forecasting (Hong et al., 2007), and others (Busse et al., 2013; DeHaan et al., 2015).

Third, the study contributes to this literature by demonstrating that mobile entertainment apps can negatively impact productivity. Previous studies showed that the use of mobile phones makes it difficult to perform daily routine activities (Liebherr et al., 2020; Chu et al., 2021). In contrast, some studies showed that smartphone apps are positively used for education and health monitoring.

This study focused on entertainment apps, which are widely acknowledged as attention-diverting and widely used (Bayer et al., 2016; Long et al., 2016). In this study, we expanded the discussion to investment decisions and proposed that the attention distraction caused by entertainment apps results in poor investment decisions. To the best of our knowledge, the potential adverse effect of entertainment apps has not been empirically tested in real-life decision-making with real financial consequences.

## 2. Materials and methods

### 2.1. General framework

We used investment data from a P2P lending platform to perform empirical analyses. The dataset provided comprehensive investment records and a wealth of information about^2^ loans, borrowers, and investor-level characteristics. In 2015, a questionnaire survey was conducted on a P2P lending platform to gather information about mobile applications used by investors. The survey included questions about the number of apps from different categories that the respondents had downloaded after registering on the platform's website. These app categories comprised social networking, photography and video, entertainment, games, health and fitness, finance, education, tools, and newspapers and magazines. By counting the total number of downloaded entertainment apps, we could identify investors who were more likely to have limited attention.^3^

We should note that the database app information was not time specific, and the information was acquired when the investors completed the questionnaire after registration in 2015 on the platform. Therefore, given the exact registration date, we restricted the data to investments made within 1 year after registration in 2015 by the respondents to balance the sample size and the change in the app list. Park et al. (2018) found that smartphone users face time, mental, privacy, and emotional barriers when attempting to delete installed apps, thus leading to hesitation to delete even unused apps. However, popular downloaded entertainment apps, such as TikTok and WeChat, were all available online before 2015. Therefore, a one-year window period was a suitable choice. The results using half-year and three-month periods were also included in the robustness checks. The data used in the study consisted of 54,206 bids, with 35,175 in 2015, 19,031 in 2016, 10,132 loans, 13,280 investors, and 3,950 borrowers.

### 2.2. Measurement of limited attention

As a proxy measure of limited attention, we used the number of entertainment apps installed in the investor's smartphone, *#Enter App*. Entertainment apps comprise a wide range of interactive activities, from purely leisure apps (music and playing games) to communication apps (social media, streaming media, instant messaging)^4^ We defined entertainment apps as those in the categories of social networking, photography, video, games,^5^ and entertainment. We counted the total number of entertainment apps downloaded to measure limited attention. Social network apps enable users to stay in touch with their friends and family. Examples of social network apps include Facebook, Instagram, and WeChat. Photography and video apps such as TikTok and Instagram are used to create, share, and find photos and short videos. Entertainment apps, such as Netflix and Amazon Prime Video, offer a wide selection of movies, TV shows, and documentaries for streaming. These types of apps are driven by excitement, curiosity, and a desire to stay connected, which often result in substantial engagement from investors. As seen in Table 1, investors tend to install a significant number of entertainment apps on their phones, with an average of 15 apps (ranging from a maximum of 91). As a result, we used the natural logarithmic value of the number of entertainment apps in our regression, denoted as *Ln (#Enter App)*.

**Table 1:** Descriptive statistics.

	**Descriptive statistics of main testing variables**							
	**N**	**Min**	**Mean**	**Median**	**Max**	**Std**.	**Skewness**	**Kurtosis**
Default	10,132	0	0.3552	0	1	0.4786	0.6051	1.3661
IRR	10,132	−1	−0.2244	0.1102	0.3449	0.5596	−0.6198	1.4368
#Enter app	13,280	1	15.3124	14	91	9.6694	1.0624	4.5402
Loan amount	10,132	100	5,180.8589	3,600	440,000	8,436.5945	24.4658	981.3721
Interest	10,132	6	17.2771	20	30	5.2328	−0.5432	2.1054
Rating	10,132	1	4.1047	5	8	1.5521	−0.4333	1.8886
Duration	10,132	1	8.3731	8	24	3.3300	0.1327	2.8757
#Listing	10,132	1	16.1776	5	521	50.4394	6.6573	51.8930
#Borrow balance	10,132	100	83,333.5872	18,449	2,557,308	285,003.3103	5.5900	35.9549
#Late pay ≤ 15	10,132	1	22.8727	7	982	86.1017	8.6026	83.2054
#Late pay > 15	10,132	0	0.0095	0	8	0.1599	24.9763	812.4138
Credit report verification	3,950	0	0.0469	0	1	0.2115	4.2858	19.3682
Face verification	3,950	0	0.8073	1	1	0.3944	−1.5586	3.4291
Borrower-young	3,950	0	0.8112	1	1	0.3914	−1.5906	3.5300
Borrower-men	3,950	0	0.7119	1	1	0.4529	−0.9360	1.8761
Borrower-married	3,950	0	0.5734	1	1	0.4946	−0.2967	1.0881
Borrower-college	3,950	0	0.8007	1	1	0.3995	−1.5056	3.2669
Borrower-employee	3,950	0	0.3718	0	1	0.4833	0.5305	1.2815
Borrower-private owner	3,950	0	0.3391	0	1	0.4735	0.6796	1.4618
Borrower-other job	3,950	0	0.2891	0	1	0.4534	0.9306	1.8660
Borrower-has home	3,950	0	0.5029	1	1	0.5000	−0.0115	1.0001
Investor-young	13,280	0	0.4782	0	1	0.4995	0.0872	1.0076
Investor-men	13,280	0	0.7097	1	1	0.4539	−0.9239	1.8535
Investor-married	13,280	0	0.2784	0	1	0.4482	0.9890	1.9781
Investor-college	13,280	0	0.3279	0	1	0.4695	0.7333	1.5377
Investor-employee	13,280	0	0.2081	0	1	0.4060	1.4382	3.0683
Investor-private owner	13,280	0	0.0371	0	1	0.1891	4.8960	24.9709
Investor-other job	13,280	0	0.7548	1	1	0.4302	−1.1844	2.4028
Investor-has home	13,280	0	0.1728	0	1	0.3781	1.7310	3.9965

This table reports descriptive statistics of the indicator variables for limited attention (*#Enter App*), investment outcomes (*Default, IRR*), and the characteristics of loans, borrowers, and investors. *Default* is set to 1 if the borrower does not pay in time and 0 otherwise; *IRR* is calculated using principle and each payment's amount and time; *#Enter App* is the total number of Social Network, Photography, and Video, Game, and Entertainment app. Loan characteristics include loan amount (in RMB), interest rate, credit rating (from 1 to 8, corresponding to AAA, AA, A, B, C, D, E & F with low to high credit risk), loan duration (in months), the number of previous borrow listing and borrow balance, the number of previous late payments that repay in 15 days, and replay more than 15 days. Characteristics of borrowers include whether the borrower has uploaded an official credit report, whether the borrower has been approved by face verification, age (young = 1 if <35 years old when placing the loans, and young = 0 otherwise), gender (male = 1; female = 0), marriage status, home status (home = 1 if has its own house, and 0 otherwise), education (college = 1 if the borrower's education is higher than college, and 0 otherwise), and occupation (employee = 1 if the occupation is employee, private owner = 1 if the occupation is private business owner, other jobs = 1 if the occupation is online seller, freelancer or others). Characteristics of investors include age, gender, marriage status, home status, education, and occupation, and all are defined in the same way as borrowers' characteristics.

#### 2.2.1. Measurement of investment performance

Limited attention is known to lead to decision-making errors with negative consequences. In the context of online lending, these errors manifest as inferior financial performance, such as increased loan defaults and poor investment returns. Therefore, to measure this impact, we created an indicator variable, *Default*, which was set to 1 for borrowers of mature loans who failed to make full payments and zero otherwise. Additionally, we calculated the internal rate of return (*IRR*) based on the payment history, including exact payment dates and amounts.

### 2.3. Control variables

We added all the available information on the platform as control variables to the empirical tests. First, at the loan level, we controlled for the following variables: *Loan Amount* is the loan amount requested (in Chinese yuan, RMB); *interest* is the annual interest rate stated in the loan request; *rating* equals 1–8 according to borrower classification into different quality tranches, indicating lower to higher levels of risk; *duration* refers to loan duration in months; *#Listing, #Borrow Balance, #Late Pay* ≤*15 days*, and *#Late Pay* >*15 days* refer to the number of previously listed borrowings, the total amount of the borrowed balance that needs to be paid, the number of late payments within 15 days, and the number of late payments >15 days made by the borrower; *Credit Report Verification* and *Face Verification* equal 1 if the borrower has uploaded an official credit report for verification and passed face authentication and 0 otherwise. Second, at the borrower characteristics level, we defined borrowers younger than 35 years while placing each bid as a young adult (B*orrower-Young* equals 1 for young adults and 0 otherwise). We categorized borrower gender into two categories: men (represented by *Borrower-Male* = 1) and women. Similarly, borrower marital status was categorized into two groups: married (represented by *Borrower-Married* = 1) and unmarried. Borrower home status was classified into two groups: those with a private home (represented by *Borrower-Home* = 1) and those without a current private home. Borrowers' educational backgrounds were divided into two categories: higher than college level (*Borrower-College* = 1) and lower than college level. Borrower occupation was categorized into three groups: employee (represented by *Borrower-Employee* = 1), private owner (represented by *Borrower-Private Owner* = 1), and others (represented by *Borrower-Other Job* = 1 if the occupation was online seller, freelancer, or others). Third, at the investor characteristics level, we controlled for characteristics such as age, gender, marital status, home information, educational background, and occupation, in addition to constructing variables for borrower characteristics.

## 3. Empirical analysis

### 3.1. Baseline results

Table 2 shows the results of the baseline empirical analysis with investment performance variables as the dependent variables and limited attention proxy *Ln (#Enter App)* as the key independent variable. In particular, we controlled for time-fixed effects of the investment time (year-month-weekday) and clustered the standard errors at the year and individual levels to account for the correlations among bids from the same year and the same investor. The Logit regression results, with *Default* as the outcome variable, are shown in Columns 1 and 2. The coefficient for *Ln (#Enter App)* in Column 1 was 0.0220 and was statistically significant (Z-value = 3.77), suggesting that a 1% increase in the number of entertainment apps is associated with a 0.0219% increase in the default risk. Column 2 included additional control variables for personal investor characteristics. Similarly, Columns 3 and 4 showed that the coefficient for *Ln (#Enter App)* was significantly negative when *IRR* was used as the dependent variable, suggesting that investors with a higher number of entertainment apps on their mobile phones are more likely to show inferior investment performance, even after controlling for many variables.

**Table 2:** Relationship between limited attention and investment outcomes: baseline results.

	**(1)**	**(2)**	**(3)**	**(4)**
	**Default**	**Default**	**IRR**	**IRR**
Ln (#Enter App)	0.0220^***^	0.0201^***^	−0.0065^**^	−0.0069^**^
	(3.77)	(3.38)	(−2.36)	(−2.47)
Loan amount	−0.1014^***^	−0.0998^***^	0.0293^***^	0.0290^***^
	(−9.29)	(−9.09)	(9.90)	(9.75)
Interest	0.0050	0.0043	0.0155^***^	0.0156^***^
	(1.39)	(1.20)	(17.84)	(17.91)
Rating	0.0393^***^	0.0399^***^	−0.0295^***^	−0.0297^***^
	(3.97)	(4.01)	(−11.35)	(−11.38)
Duration	0.0898^***^	0.0893^***^	−0.0184^***^	−0.0182^***^
	(41.76)	(41.26)	(−32.25)	(−31.77)
#Listing	−0.6056^***^	−0.6054^***^	0.1316^***^	0.1315^***^
	(−36.15)	(−35.98)	(31.90)	(31.72)
#Borrow balance	−0.1320^***^	−0.1318^***^	0.0225^***^	0.0225^***^
	(−9.36)	(−9.31)	(6.13)	(6.09)
#Late pay ≤ 15	0.5252^***^	0.5244^***^	−0.1178^***^	−0.1176^***^
	(55.60)	(55.44)	(−53.92)	(−53.88)
#Late pay>15	0.3845^***^	0.3971^***^	−0.0812^***^	−0.0839^***^
	(4.97)	(4.98)	(−4.97)	(−5.03)
Credit report verification	−0.2428^***^	−0.2413^***^	0.0559^***^	0.0554^***^
	(−10.95)	(−10.83)	(9.95)	(9.79)
Face verification	−0.3071^***^	−0.3085^***^	0.0774^***^	0.0776^***^
	(−18.17)	(−18.16)	(16.32)	(16.27)
Borrower-young	0.2951^***^	0.2926^***^	−0.0717^***^	−0.0711^***^
	(15.66)	(15.47)	(−14.48)	(−14.30)
Borrower-men	−0.2388^***^	−0.2419^***^	0.0616^***^	0.0623^***^
	(−14.50)	(−14.64)	(13.35)	(13.45)
Borrower-married	−0.1031^***^	−0.1038^***^	0.0280^***^	0.0282^***^
	(-6.61)	(−6.63)	(6.49)	(6.52)
Borrower-college	0.1524^***^	0.1490^***^	−0.0473^***^	−0.0467^***^
	(7.33)	(7.14)	(−8.50)	(−8.36)
Borrower-private owner	−0.0591^***^	−0.0610^***^	0.0293^***^	0.0298^***^
	(−3.58)	(−3.68)	(6.33)	(6.43)
Borrower-other job	−0.1767^***^	−0.1774^***^	0.0424^***^	0.0427^***^
	(−8.90)	(−8.90)	(8.07)	(8.09)
Borrower-has home	−0.2783^***^	−0.2786^***^	0.0597^***^	0.0599^***^
	(−18.29)	(−18.24)	(14.59)	(14.57)
Investor-young		−0.0182		0.0051
		(−1.18)		(1.25)
Investor-men		0.0343^**^		−0.0026
		(2.07)		(−0.58)
Investor-married		0.0202		−0.0023
		(0.92)		(−0.39)
Investor-college		−0.0389		0.0053
		(−1.58)		(0.79)
Investor-private owner		0.0073		0.0043
		(0.24)		(0.52)
Investor-other job		−0.0255		0.0025
		(−1.24)		(0.44)
Investor-has home		0.0308		−0.0073
		(1.46)		(−1.28)
Constant	1.0418^***^	1.0474^***^	−0.8291^***^	−0.8300^***^
	(8.79)	(8.60)	(−27.01)	(−26.21)
Time fixed effect	YES	YES	YES	YES
Observations	54,206	54,206	54,206	54,206
Pseudo/Adjusted R^2^	0.1190	0.1193	0.1002	0.1003

This table reports regression results with the limited attention proxy *Ln(#Enter App)* as the independent variable and investment outcome proxy *Default* and *IRR* as the dependent variables, with or without controlling for investor characteristic variables. Time-fixed effect of investing time (year-month-weekday) is controlled. *Z*-values and *T*-values are clustered at investor and invest year level and reported in parentheses.

^*^, ^**^, and ^***^ indicate statistical significance at 10, 5, and 1% levels, respectively.

### 3.2. Addressing endogeneity issue

#### 3.2.1. DID analysis

Through manual information collection, there were seven large-scale exogenous events in 2015, during which the Internet service of a large entertainment company went down unexpectedly, including games, social media, video watching, and entertainment servers.^6^ We keep the investments of investors who have installed the seven apps, and constructed the variable *Event*, which equals one for the investments made on the seven outage days, and zero for investments made 1 day earlier. The results of DID with multiple time periods in Table 3 show that the events significantly reduce the likelihood of default and increase the rate of return. The occurrence of internet outrage events results in a decrease in the usage of mobile entertainment apps, reducing the limited attention caused by these apps. On the day of such events, investment performance tends to improve compared to the performance observed 1 day earlier due to the attenuation of limited attention. Therefore, it was confirmed that entertainment apps have attention-diverting effects and may lead to investors making poor investments.

**Table 3:** Addressing the endogeneity problem.

	**(1)**	**(2)**	**(3)**	**(4)**	**(5)**
	**Default**	**IRR**	**Ln(#Enter App)**	**Default**	**IRR**
Event	−0.7103^***^	0.1122^**^			
	(−2.82)	(2.50)			
Ln (#Enter App)				0.1133^**^	−0.0526^**^
				(1.96)	(−1.96)
Search index			0.0049^**^		
			(2.35)		
Ln (Avg.#Enter App)			1.0923^***^		
			(32.13)		
Control	YES	YES	YES	YES	YES
Constant	22.2410^***^	−2.6241^***^	−0.4340^***^	0.4164^***^	−0.7299^***^
	(6.62)	(−7.16)	(−4.80)	(2.82)	(−10.87)
Time fixed effect	YES	YES	YES	YES	YES
Observations	2,052	2,052	54,206	54,206	54,206
Pseudo/adjusted R^2^	0.3083	0.2694			0.0971

This table reports DID and IV results to address the endogeneity problem. *Event* equals one for investments made on the seven event days for investors who have installed the corresponding app and zero for investments made 1 day earlier. *Search index* is the sum of the city-level Baidu Index of the listed keywords in 2015, including mobile social apps, mobile video apps, mobile game apps, and mobile entertainment apps. *Avg.#Enter App* is the average number of downloaded entertainment apps at the same age and gender as the investor (excluding the investor). Column 3 report the first stage result, and Column 4 and 5 report the second stage results. All regressions control characteristics of loans, borrowers, and investors and the time-fixed effect. Z-values and *T*-values are clustered at investor and invest year level and reported in parentheses.

^*^, ^**^, and ^***^ indicate statistical significance at 10, 5, and 1% levels, respectively.

#### 3.2.2. Instrumental variable

We further employed an instrumental variable approach to address potential endogeneity concerns. Specifically, we used the sum of the city-level Baidu Index^7^ for the following keywords in 2015: “mobile social app,” “mobile video app,” “mobile game app,” and “mobile entertainment app” as the first instrumental variable (IV). We also employed the average number of entertainment apps downloaded by individuals with the same age and gender as the investor (excluding the investor) as another IV. The theoretical rationale behind this approach is that the search index is widely used to measure public attention and interest (Swamy and Dharani, 2019). A higher search frequency indicates a higher likelihood of downloading many entertainment-related apps. In addition, individuals of the same age and gender tend to have similar entertainment preferences (Stachl et al., 2017), leading them to download similar entertainment apps. The results of the first stage regression in Column 3 reveal a significant positive relationship between the two instrumental variables and *Ln (#Enter App)*. The Cragg-Donald-Wald F statistic of 515.86 passes the weak identification tests, suggesting the relevance of the instrumental variables. Moreover, the Hansen J statistic of 2.616 with a *p*-value of 0.1061 rejects the overidentification test, demonstrating the exogeneity of the instrument variables. The second-stage results reinforce the main findings, with the coefficient being consistent with them.

#### 3.2.3. Cross-sectional analysis

We also conducted two sets of cross-sectional studies to address the alternative explanations. First, investors tend to suffer more from limited attention on Friday when using entertainment-related apps for weekend schedule arrangements. We constructed an indicator variable on *Friday* and examined its interaction with *Ln(#Enter App)*. Column 1 of Table 4 shows that the interaction term coefficient with *Default* as the dependent variable was significantly positive and was negative with *IRR* as the dependent variable (column 2), suggesting a more potent effect of the number of entertainment apps on investment performance on Friday.

**Table 4:** Cross-sectional analysis.

	**(1)**	**(2)**	**(3)**	**(4)**
	**Default**	**IRR**	**Default**	**IRR**
Ln (#Enter App)	0.0092	−0.0017	0.0091	−0.0011
	(0.64)	(−0.50)	(1.15)	(−0.79)
Ln (#Enter App) ^*^ Friday	0.0692^**^	−0.0150^**^		
	(2.36)	(−2.20)		
Friday	−0.1230	0.0261		
	(−1.53)	(1.41)		
Ln (#Enter App) ^*^ High speed			0.0238^***^	−0.0058^**^
			(3.33)	(−3.32)
High speed			−0.0398	0.0108
			(−1.17)	(1.33)
Constant	0.6630^***^	−0.7325^***^	0.5256^**^	−0.7148^***^
	(4.40)	(−21.14)	(2.49)	(−14.26)
Control	YES	YES	YES	YES
Time fixed effect	YES	YES	YES	YES
Observations	31,960	31,960	54,206	54,206
Pseudo/Adjusted R^2^	0.0962	0.0940	0.1348	0.1261

This table reports the results of *#Enter App* and *Default, IRR* with different interaction terms. *Friday* equals one if the investor places an investment on Friday, and zero for Mondy to Thursday. *High Speed* equals one if the local internet speed is higher than the national average in the same year and zero otherwise. All regressions control characteristics of loans, borrowers, and investors and time-fixed effect. Z-values and standard errors are clustered at investor and invest year level and reported in parentheses.

^*^, ^**^, and ^***^ indicate statistical significance at 10, 5, and 1% levels, respectively.

In addition, nothing is more frustrating than a slow mobile data connection, especially when attempting to stream a video or play a game. Therefore, entertainment-related apps may cause a higher degree of limited attention when accessed on high-speed Internet due to the enhanced user experience and memory. To test this, we included an interaction term between *Ln (#Enter App)* and *High Speed*, which was defined as local internet speeds that are higher than the national average for the same year, in our baseline regression. The results are consistent with our predictions (columns 3 and 4, Table 4).

### 3.3. Differentiating entertainment app category

We separately examined the effect of four types of entertainment apps on investment performance using responses to the question. Table 5^8^ shows that a higher number of downloads for each type was associated with a higher default rate and a lower investment return. A comparison of the magnitude of the coefficients showed that games and entertainment apps have a more substantial effect on attentional distraction. However, the seemingly unrelated estimation test revealed no significant difference between the coefficients of the four types of apps. Therefore, the total number of entertainment-related apps is significant for investment performance, and there is no substantial difference between social, photography, entertainment, and game apps.

**Table 5:** Relationships between different categories of entertainment apps and investment outcomes.

	**(1)**	**(2)**	**(3)**	**(4)**	**(5)**	**(6)**	**(7)**	**(8)**
	**Default**				**IRR**			
Ln (#Social App+1)	0.0254^***^				−0.0029			
	(2.85)				(−1.41)			
Ln (#Photography App+1)		0.0254^***^				−0.0038^*^		
		(2.73)				(−1.76)		
Ln (#Entertainment App+1)			0.0263^***^				−0.0043^**^	
			(3.47)				(−2.43)	
Ln (#Game App+1)				0.0272^***^				−0.0040^*^
				(2.68)				(−1.68)
Control	YES	YES	YES	YES	YES	YES	YES	YES
Constant	1.0525^***^	1.0617^***^	1.0530^***^	1.0663^***^	−0.8345^***^	−0.8351^***^	−0.8335^***^	−0.8359^***^
	(8.64)	(8.72)	(8.65)	(8.76)	(−31.01)	(−31.05)	(−31.00)	(−31.10)
Time fixed effect	YES	YES	YES	YES	YES	YES	YES	YES
Observations	54,206	54,206	54,206	54,206	54,206	54,206	54,206	54,206
Pseudo/Adjusted R^2^	0.1193	0.1193	0.1193	0.1193	0.1047	0.1047	0.1047	0.1047

This table reports regression results with the number of each four types of apps as the independent variable and investment outcome proxy *Default* and *IRR* as the dependent variables. All regressions control characteristics of loans, borrowers, and investors and the time-fixed effect. *Z*-values and *T*-values are clustered at investor and invest year level and reported in parentheses.

^*^, ^**^, and ^***^ indicate statistical significance at 10, 5, and 1% levels, respectively.

## 4. Mechanism analyses

### 4.1. Information process

A widely recognized feature of limited attention is that it can cause investors to partially neglect information, leading to underreaction and overreaction to earnings announcements (Hirshleifer et al., 2011) and the allocation of more attention to more salient information (Frydman and Wang, 2020). The operating mechanism of the online P2P market requires borrowers to post a loan title, a short description of themselves, the intended use of the loan, and the source of payment. Therefore, if a borrower uploads a longer description, investors may overlook important information and make poor investment decisions if they are distracted by entertainment-related apps. To test this hypothesis, we created two variables: *Long Title* and Long Description. The variable *Long Title* was defined as one of the numbers of words in the loan title being greater than 30 (median value: 29.667) and zero otherwise. The variable *Long Description* was defined as one if the number of words in the loan description was >84 (median value: 83.979). The results, shown in Table 6, indicate that the interaction term between *Ln(#Enter App)* and *Long Title* was positive and significant for *Default* (column 1) and negatively significant for *IRR* (column 2). We found similar results using the *Long Description* variable.

**Table 6:** Mechanism analysis: from the perspective of the information process.

	**(1)**	**(2)**	**(3)**	**(4)**
	**Default**	**IRR**	**Default**	**IRR**
Ln (#Enter App)	0.0133	−0.0028	0.0211^*^	−0.0043
	(1.03)	(−0.90)	(1.83)	(−1.62)
Ln (#Enter App) ^*^ Long title	0.0725^***^	−0.0129^**^		
	(2.74)	(−2.34)		
Long title	−0.2383^***^	0.0462^***^		
	(−3.38)	(3.17)		
Ln (#Enter App) ^*^ Long description			0.2039^**^	−0.0491^**^
			(2.02)	(−2.04)
Long description			2.5839^***^	−0.5258^***^
			(9.93)	(−8.39)
Control	YES	YES	YES	YES
Constant	1.0759^***^	−0.8379^***^	3.4007^***^	−1.2276^***^
	(7.61)	(−26.20)	(21.64)	(−39.30)
Time fixed effect	YES	YES	YES	YES
Observations	54,206	54,206	54,206	54,206
Pseudo/Adjusted R^2^	0.1049	0.1006	0.1203	0.1156

This table reports the results of *Ln(#Enter App)* and *Default, IRR* with different interaction terms from the perspective of the information process. *Long Tile* equals 1 if the number of words in the borrowing title is larger than 30 and 0 otherwise. *Long Description* equals 1 if the number of words in the borrowing description is larger than 84 and 0 otherwise. All regressions control characteristics of loans, borrowers, and investors and the time-fixed effect. *Z*-values and *T*-values are clustered at investor and invest year level and reported in parentheses.

^*^, ^**^, and ^***^ indicate statistical significance at 10, 5, and 1% levels, respectively.

### 4.2. Familiarity bias

Extensive research on heuristics and biases, originating primarily in psychology, has shown that people often resort to simple cognitive shortcuts when processing information, resulting in systematic biases in decision-making. Kahneman (2011) proposed that attention limitations can trigger a shift from slow, deliberate thinking to fast, intuitive thinking, which relies on numerous heuristics. Gennaioli and Shleifer (2010) explored the concept of intuitive inference, which automatically combines external information with internally retrieved information from memory. They found that this type of inference accounts for some judgment biases, including familiarity bias. Jacoby et al. (1989) found that divided attention does not affect the gain of familiarity, as assessing familiarity requires minimal attention. Given this information, we assumed that, when attention is limited, investors are more likely to rely on familiarity heuristics; therefore, limited attention caused by the use of entertainment apps may have a substantial impact on investment returns. Additionally, we constructed two variables to examine the influence of familiarity on investment. These variables are *Same Province*, which was set to one if the investor was born in the same province as the borrower and zero otherwise. The other variable was *Invest Before*, which was set to one if the investor had previously lent money to the same borrower and zero otherwise. The results presented in Table 7 indicate that, for investors who had a sense of familiarity with the borrower, attention distraction caused by entertainment apps had a more negative effect on investment performance, supporting our hypothesis.

**Table 7:** Mechanism analysis: from the perspective of familiarities bias.

	**(1)**	**(2)**	**(3)**	**(4)**
	**Default**	**IRR**	**Default**	**IRR**
Ln (#Enter App)	0.0339^***^	−0.0071^***^	0.0128^**^	−0.0031^**^
	(3.10)	(−2.81)	(2.20)	(−2.26)
Ln (#Enter App) ^*^ Same Province	0.5648^*^	−0.1421^**^		
	(1.68)	(−1.98)		
Same Province	−1.3665	0.3457^*^		
	(−1.56)	(1.95)		
Ln (#Enter App) ^*^ Invest before			0.0593^**^	0.0036
			(2.37)	(0.84)
Invest before			−0.2778^***^	0.0067
			(−4.55)	(0.71)
Control	YES	YES	YES	YES
Constant	1.1954^***^	−0.8482^***^	1.0592^***^	−0.8316^***^
	(8.96)	(−28.12)	(8.67)	(−30.81)
Time fixed effect	YES	YES	YES	YES
Observations	41,537	41,537	54,206	54,206
Pseudo/Adjusted R^2^	0.1014	0.1013	0.1197	0.1048

This table reports the results of *Ln(#Enter App)* and *Default, IRR* with different interaction terms from the perspective of familiarities bias. *Same Province* equals 1 if the investor and borrower are from the same province and 0 otherwise. *Invest Before* equals 1 if the investor has invested the borrower's listing before and 0 otherwise. All regressions control characteristics of loans, borrowers, and investors and the time-fixed effect. *Z*-values and *T*-values are clustered at investor and invest year level and reported in parentheses.

^*^, ^**^, and ^***^ indicate statistical significance at 10, 5, and 1% levels, respectively.

## 5. Robustness tests

We conducted several robustness tests (see Table 8). We first examined the window period of half a year and 3 months after the investors registered on the platform. The app information was recorded at the time of registration in 2015 and was not updated. This resulted in measurement noise for the recorded data for a substantial amount of time after 2015. Therefore, to mitigate the effect of changes in the number of mobile apps, we restricted the sample to half a year and 3 months after registration. Despite the potential measurement noise being excluded, the effect remained significant (columns 1–4). We then examined any possible influences resulting from the empirical methods used. We used a linear probability model to test the relationship between *Ln (#Enter App)* and *Default*; the coefficient remained positive and significant (column 5). To determine if the effect was due to the total number of installed apps, we also included the natural logarithmic value of the total number of apps as a control in the baseline regression *Ln (#Total App)*. The coefficients for the primary variable showed little change. In contrast, the *Ln (#Total App)* coefficient was not significant, suggesting that attention is mainly affected by specific types of apps (e.g., entertainment apps) instead of all kinds (Columns 6 and 7).

**Table 8:** Robustness tests.

	**(1)**	**(2)**	**(3)**	**(4)**	**(5)**	**(6)**	**(7)**
	**Default**	**IRR**	**Default**	**IRR**	**Default**	**Default**	**IRR**
	**Subsample in half-year since registration**		**Subsample in 3 months since registration**		**LPM model**	**Control total number of apps**	
Ln (#Enter App)	0.0274^***^	−0.0035^*^	0.0272^***^	−0.0057^**^	0.0047^**^	0.0368^**^	−0.0075^**^
	(3.49)	(−1.98)	(2.67)	(2.32)	(2.10)	(2.45)	(−2.13)
Ln (#Total App)						−0.0824	0.0150
						(−0.88)	(0.65)
Control	YES	YES	YES	YES	YES	YES	YES
Constant	1.1026^***^	−0.8517^***^	1.1390^***^	−0.8211^***^	0.7631^***^	1.0263^***^	−0.8287^***^
	(6.67)	(−23.60)	(5.22)	(16.61)	(27.80)	(7.28)	(−25.98)
Time fixed effect	YES	YES	YES	YES	YES	YES	YES
Observations	20,157	20,157	12,600	12,600	54,206	54,206	54,206
Pseudo/adjusted R^2^	0.1386	0.1134	0.1633	0.0957	0.1221	0.1048	0.1004

Columns 1 and 2 are based on the investments that the investor made within the half year after registration, and Columns 3 and 4 are within 3 months; Columns 5 use the LPM model to test the relationship between *Ln(#Enter App)* and *Default*; Colum 6 and 7 control for the total number of mobile apps of the investor, *Ln(#Total App)*. All regressions control characteristics of loans, borrowers, and investors and the time-fixed effect. *Z*-values and *T*-values are clustered at investor and invest year level and reported in parentheses.

^*^, ^**^, and ^***^ indicate statistical significance at 10, 5, and 1% levels, respectively.

## 6. Conclusion

Psychologists and behaviorists have recently become interested in the widespread possession of mobile phones, demonstrating that it is crucial in understanding people's emotions, behaviors, and psychological wellbeing. Our study delves into this area by investigating the impact of attention-distracting entertainment apps on financial market investment performance. By analyzing real financial market investment data, we discovered that limited attention resulting from the use of entertainment-related apps is a key determinant of poor investment performance. We found that limited attention increases the likelihood of defaults and lower returns. To further prove the impact of limited attention, we used an exogenous natural experiment of entertainment service unavailability and found that outage can enhance investment performance. Moreover, results from the instrumental variable were also consistent. The mechanism analysis showed that lower-performance bidding behaviors were subject to information neglect and familiarity heuristics. The evidence is consistent with previous findings that limited attention has adverse effects and leads to cognitive shortcuts, thereby uncovering the potential underlying processes behind inattentive thinking.

Our study provides insights into economic agents' cognitive behaviors when they process information, especially when their cognitive resources are constrained. Although we do not delve into all the mental processes involved in economic decision-making under limited attention, future research should further explore this area thoroughly. Incorporating the cognitive effects of mobile phones into both theoretical and empirical analyses of financial markets would be a promising direction for future research. In addition, comprehensive data on individual mobile phone usage will greatly improve the research framework.

## Data availability statement

The raw data supporting the conclusions of this article will be made available by the authors, without undue reservation.

## Author contributions

YZ and YL contributed to conception and design of the study. YD organized the database. YZ and YD performed the statistical analysis. YZ wrote the first draft and revised draft of the manuscript. All authors contributed to manuscript revision, read, and approved the submitted version.

## Footnotes

^1^According to the 47th China Statistical Report on Internet Development released by the China Internet Network Information Center (CINIC), 99.7% of Internet users had access to the Internet *via* mobile phones, and there were 986 million mobile users at the end of 2020. In addition, the average Internet usage time was approximately 26.2 h per week.

^2^The P2P lending platform is one of China's largest and most active platforms. It mainly provides services to individual borrowers who have poor credit, no access to traditional lenders, and live in small cities and towns. The borrowing amount is RMB¥ 5,000 on average to meet consumption needs.

^3^The respondents answered the questions in the survey based on their subjective recall. Psychology researchers discovered that respondents tend to retrieve information associated with strong ties, intense interactions, and high closeness (Brewer, 2000; Töpfer and Hollstein, 2021). The frequently used apps have strong interactions and connections, with the investor, while the rarely used apps may be easily forgotten when answering the survey. Therefore, the number of entertainment-related apps downloaded, as reported in the survey, includes usage information and serves as a suitable proxy for limited attention.

^4^ https://www.consumeracquisition.com/faq/what-are-entertainment-apps

^5^There could be some game apps, such as the “puzzle game,” that seem to be designed to improve cognitive activities. We searched these apps on the website and discovered that they all have many levels of gameplay and are developed by a game company instead of nonprofit organizations or research institutions solely for cognitive training. In this way, these game apps share the typical characteristics of a game app with a sense of excitement and achievement linked to striving for high scores and can distract attention.

^6^The seven exogenous events in 2015 included the service outage of Tanke World, a game app, on 9 February, Douban on 21 April, Zhihu on 15 June, IQiyi on 3 July, QQ on 6 August, Leshi on 19 September, and WeChat on 6 November. The survey also asks for the installation information of several popular apps.

^7^Baidu Index was obtained from the website of “Baidu Index” (http://index.baidu.com/), which shows the search volume of Baidu's, the largest search engine in China, using specific keywords at different time periods and cities. Besides, we use the mobile-terminal index as IV instead of the PC terminal index.

^8^As the number of downloaded apps of each type could be zero for some investors, we use the natural logarithmic value of the number plus one.
